# Deciphering Squamous Cell Carcinoma Using Multidimensional Genomic Approaches

**DOI:** 10.1155/2011/541405

**Published:** 2010-12-27

**Authors:** Ewan A. Gibb, Katey S. S. Enfield, Ivy F. L. Tsui, Raj Chari, Stephen Lam, Carlos E. Alvarez, Wan L. Lam

**Affiliations:** ^1^Department of Integrative Oncology, British Columbia Cancer Agency Research Centre, Vancouver BC, Canada V5Z 1L3; ^2^Department of Pediatrics, Center for Molecular and Human Genetics, The Research Institute at Nationwide Children's Hospital, Columbus, OH 43205, USA

## Abstract

Squamous cell carcinomas (SqCCs) arise in a wide range of tissues including skin, lung, and oral mucosa. Although all SqCCs are epithelial in origin and share common nomenclature, these cancers differ greatly with respect to incidence, prognosis, and treatment. Current knowledge of genetic similarities and differences between SqCCs is insufficient to describe the biology of these cancers, which arise from diverse tissue origins. In this paper we provide a general overview of whole genome approaches for gene and pathway discovery and highlight the advancement of integrative genomics as a state-of-the-art technology in the study of SqCC genetics.

## 1. Introduction

Squamous cell carcinoma (SqCC) initiation and development is characterized by the accumulation of genetic alterations. Over the past two decades, technological advances used to identify these alterations have expanded from single-gene queries to genome-wide methods of analysis in a multitude of dimensions, including genomics, epigenomics, and transcriptomics. Mutations and copy number alterations at the DNA level represent genomic alterations, while aberrant methylation patterns and histone modifications reflect epigenetic changes. Gene expression changes manifest either as a direct consequence of genetic and epigenetic alterations or as reactive changes and downstream effects. Furthermore, transcription patterns are also mediated by noncoding RNAs such as microRNAs (miRNAs), which can be deregulated by the aforementioned genetic and epigenetic alterations. The global profiling of each omics dimension represents a remarkable technological and bioinformatic achievement. However, the integration of these individual dimensions must be pursued in order to provide a comprehensive view of the impact of gene disruption in SqCC. Hence, there is a growing need to merge these multiple dimensions of data to identify concerted and complementary alterations that lead to the perturbation of oncogenic pathways and gene networks.

## 2. Multidimensional Analysis of Cancer Genomes

### 2.1. Genomic Alterations in Cancer

**Figure 1:**
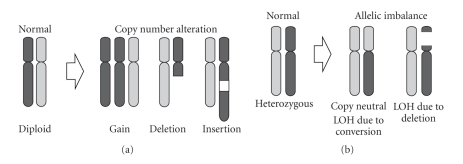
Mechanisms of DNA copy number alteration. (a) Segmental gains and losses can lead to DNA copy number alterations. (b) Allelic imbalance and loss of heterozygosity (LOH) can arise from a deletion event or gene conversion during mitosis.

Genome destabilization is one of the hallmarks of cancer and is reflected by the accumulation of multiple genetic alterations such as chromosomal translocation, DNA copy number alteration, and sequence mutation [1]. Chromosomal translocation has been shown activate oncogenes by gene fusion [2–4]. Segmental duplication and amplification leads to increased gene dosage and, often, inappropriate expression of oncogenes [5] (Figure 1). Deletion leads to loss of tumor suppressor function through either haploinsufficiency or two-hit inactivation of functional alleles [6, 7]. Examples of such two-hit mechanisms are homozygous deletion or a combination of deletion and gene mutation. DNA mutation can lead to a variety of effects, such as constitutive gene activation or inactivation. A variety of technology platforms have been developed that are tailored to the detection of specific types of genomic alterations. These technologies are summarized in Table 1.

### 2.2. Epigenomics of Cancer

**Figure 2:**
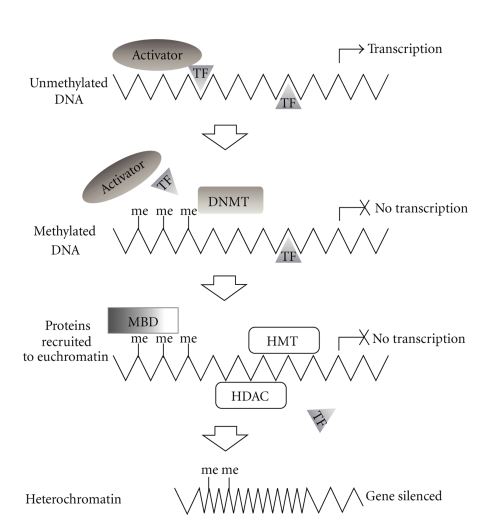
Mechanisms of epigenetic gene silencing. Unmethylated DNA is accessible to activator proteins and transcription factors (TF) enabling transcription. DNA methylation by DNA methyl transferases (DNMT) causes steric inhibition of transcription complexes thus blocking transcription. Methylated DNA (me) is recognized by methyl binding domain proteins (MBD), histone deacetylases (HDAC), and histone methyltransferases (HMT) which stimulate chromatin remodeling. Compaction of DNA into condensed chromatin (heterochromatin) results in transcriptionally inactive DNA.

Epigenetics is the study of heritable chromosomal changes that influence transcription but do not directly alter the DNA sequence [24]. Mechanisms of epigenetic gene regulation include DNA methylation and histone modification. Altered epigenetic regulation in cancer includes hypo- and hypermethylation of genes, which can lead to increased genomic instability and the activation of oncogenes or the silencing of tumor suppressor genes, respectively. Similarly, the modification of histones can modulate chromatin structure and accessibility (Figure 2). The genome-wide alignment of epigenetic aberrations with genetic alterations can lead to the identification of genes disrupted in both alleles. For example, in the two-hit model one allele could be lost to deletion, while the other copy is silenced by hypermethylation. These events can in turn lead to changes in gene expression levels [25–27]. A wide range of methodologies exists for the determination of methylation status, which rely on bisulfite conversion of cytosines [28–32], immunoprecipitation of methylated DNA [33, 34], or the sensitivity of endonucleases to DNA methylation [35, 36]. The technologies in methylome analysis are summarized in Table 2 [27]. Histone modification analysis is complex due to the variety of modifications and the different residues being modified. Common methods of assessment such as ChIP-chip and ChIP-seq involve immunoprecipitation using antibodies against specific types of modification and are the subject of recent review articles [37–39].

### 2.3. Gene Expression Profiling

The downstream effect of genomic and epigenomic alterations is the dysregulation of gene expression. Global gene expression profiling has been instrumental in both disease classification and cancer gene discovery [49–54], as well as in biomarker discovery in a wide range of cancers [55–57]. The evolution of gene expression profiling technologies and their applications to cancer biology is well documented in the literature [58, 59]. While microarrays are widely used for expression profiling, sequence-based approaches such as serial analysis of gene expression (SAGE) present a digital alternative to measuring transcript abundance [60]. More recently, the emergence of massively parallel and next-generation sequencing platforms has revolutionized whole transcriptome analysis [20–23]. Expression changes are not limited to protein coding genes; alterations in miRNA levels have been well documented in various cancer types, discussed below.

### 2.4. Noncoding RNAs

Approximately 90% of the human genome is transcribed, with only a small fraction of these transcripts representing protein coding mRNAs [61–64]. The remaining transcripts are comprised of a wide range of noncoding RNAs (ncRNAs) including ribosomal RNAs (rRNAs), transfer RNAs (tRNAs), miRNAs, and a recently described class, long noncoding RNAs (lncRNAs) [65].

MicroRNAs are small ~22 nucleotide (nt) RNAs involved in posttranscriptional silencing of mRNA targets [66]. These regulatory RNAs exhibit an enormous influence on most fundamental biological processes, altering the expression of proteins by inhibiting translation or promoting mRNA degradation [67]. Aberrant miRNA expression has been linked to a range of diseases, including cancer progression and prognosis [68, 69]. Many of the mechanisms described to alter normal gene expression in cancer can also influence the expression of miRNAs. For instance, the gain or loss of a specific miRNA can function as either an oncogene or a tumor suppressor [70–72], and mutations in miRNA sequences or miRNA processing machinery can have a tremendous impact on miRNA regulatory function [73]. Intriguingly, the expression of miRNAs can also be regulated by epigenetic mechanisms [74], while in turn, miRNA expression can modulate epigenetic regulation by targeting enzymes responsible for histone modification and DNA methylation [75, 76].

More recently, lncRNAs have been described to function as epigenetic regulators of transcription, chromatin remodeling and cellular development [65, 77, 78]. While the extent of their involvement in tumorigenesis is unknown, the dysregulation of lncRNAs may prove to be yet another level of complexity in the cancer genomic landscape.

In combination with genomic, epigenomic, and transcriptomic alterations, the capacity of ncRNAs to influence the expression of a range of biological processes highlights the need to merge the multiple dimensions of whole genome analysis. Integrative genomics is the response to this requirement as highlighted below.

### 2.5. Integrative Genomics

**Figure 3:**
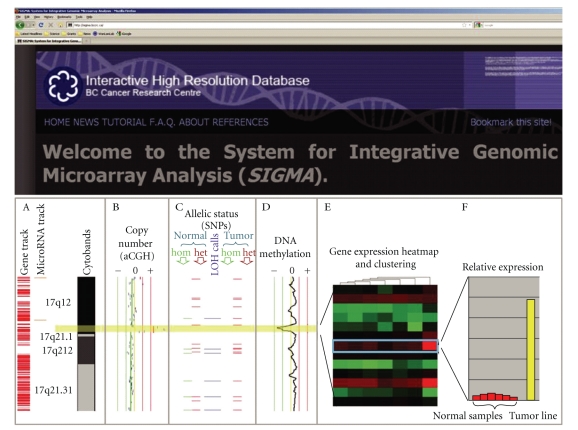
Multidimensional omics data integration using SIGMA2 software. Combined genetic, epigenetic and gene expression analysis of cancer samples facilitates identification of oncogenes and tumor suppressor genes which are concertedly disrupted. (a) Examples of annotation tracks. (b) Copy number profile from array CGH experiment—a focal DNA amplification of a region on 17q12 is highlighted in yellow. (c) Allelic status (SNP array). This region is also encompassed in a large stretch of allelic imbalance. Blue horizontal bars indicate loci that become homozygous (loss of heterozygosity) in the tumor sample. (d) DNA methylation analysis (MeDIP-microarray) shows a concurrent loss of methylation, as indicated by a peak shifted to left of the center line. (e) Heat map summary of gene expression profile in the region of interest. The gene boxed in blue on the heatmap is *ERBB2*. It shows the highest level of differential expression between the tumor line and a panel of normal tissue samples. (f) The histogram displays the relative expression of the tumor sample as compared with the normal samples for *ERBB2* (i.e., expanded view from heat map).

Until recently, the study of cancer genomes, epigenomes, and transcriptomes has largely been done in a solitary manner. With technologies to assess these different dimensions becoming more accessible (Figure 3), the integration of these diverse data dimensions in parallel is the next logical step. In fact, large-scale initiatives, such as those led by The Cancer Genome Atlas (TCGA) Research Network (http://cancergenome.nih.gov/), are proposing the comprehensive characterization of genomic, epigenomic, and transcriptomic alterations for a multitude of cancer types. 

While the majority of previous integrative studies have focused on correlating copy number alterations with gene expression changes, there have been a few more recent studies which have incorporated the epigenomic dimension as well [79–83]. Moreover, from these studies, it is clear that both quantitative and qualitative benefits are reaped when employing a Multidimensional approach. For example, the abilities to (i) associate more of the observed aberrant gene expression to genetic and epigenetic alterations (ii) identify complementary alterations in different samples which lead to similar downstream effects, and (iii) elucidate complex disruption patterns of both known and novel signaling pathways are some of the key findings that have been made. However, a consequence of assessing the cancer cell at a high resolution is the generation of copious amounts of data and the subsequent need for specialized bioinformatic tools [84]. As a result, there have been a number of recently developed tools to address this need.  Table 3 lists these tools and where they can be obtained.

## 3. Applying Integrative Genomics to Squamous Cell Carcinoma

The unique biology of SqCCs arising from different tissues is reflected in their underlying genetic variability. Here we examine the use of multiple dimensions of omics data in elucidating the underlying genetic features of three common types of SqCCs. (1) Oral SqCC illustrates how elevated genomic instability may predispose early lesions to cancer progression. (2) Lung SqCC has recently been found to harbor critical cell lineage-specific genetic alterations. (3) Finally, skin SqCC demonstrates how a single pathway can be disrupted at multiple nodes, and specific pathways are dysregulated in cancer subtypes. These inherent tissue-specific differences in SqCCs may yield novel genes and pathways suitable for tailoring cancer diagnostics and therapy. The use of Multidimensional, genome wide analysis will be instrumental in facilitating these discoveries.

### 3.1. Genetic Instability in Oral Squamous Cell Carcinoma

The majority of oral malignancies in the upper aerodigestive tract are SqCCs [93]. The strongest etiological factor for oral carcinogenesis is tobacco smoke, while human papillomavirus (HPV) infection has been frequently detected in nonsmoker and younger oral squamous cell carcinoma (OSCC) patients [93–97]. Differences in the pattern of genetic alterations suggest that HPV-positive oral cancers may represent a distinct disease entity that develops via a different genetic pathway [98]. 

**Figure 4:**
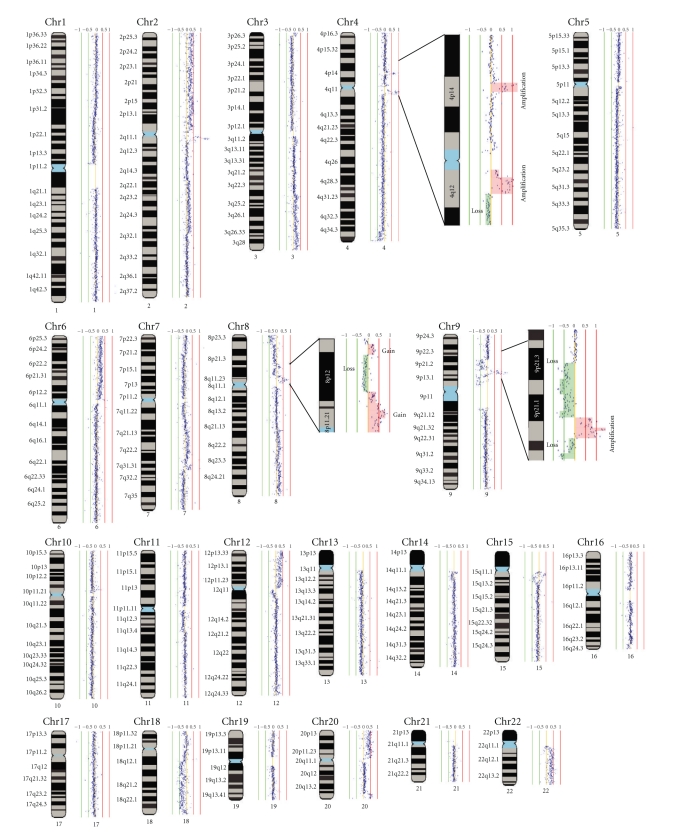
Whole-genome tiling-path array profile of an oral low-grade dysplasia. Normalized log2 signal intensity ratios were plotted using *SIGMA *software [85, 86]. Cytoband pattern corresponding to the data points was drawn to the left of each data plot. The red and green vertical lines represent signal intensity ratios from −1 to +1 with an increment of 0.5. The magnified insets (for regions of chromosome 4, 8, and 9) include segmental copy number gain highlighted in orange and copy number loss highlighted green.

As in other SqCCs, the evolution of OSCC is known to result from the acquisition of multiple genetic events targeting different genes and molecular pathways [99]. Genomic instability increases progressively from hyperplasia through various stages of dysplasia to invasive carcinoma [100]. Although specific genetic alterations have been frequently detected in early stage dysplasias, it is believed that it is the accumulation of genomic instability, rather than the sequence of specific events, that determines oral carcinogenesis [100, 101]. Thus, examining oral premalignant lesions (OPLs) would allow the identification of early genetic events that may be masked by increased genomic instability in late stage tumors and, as such, could potentially identify biomarkers for predicting OPLs that will progress to cancer. Previous studies have identified several loci, including multiple regions on chromosome arm 3p, such as the 3p14 locus that harbors *FHIT* (*Fragile Histidine Triad*), to be commonly deleted in oral dysplasias [102–104]. However, assaying only several loci does not provide sufficient predictive power for progression. Whole-genome tiling-path array comparative genomic hybridization (aCGH) facilitated comprehensive mapping of genetic alterations in premalignant and malignant oral tissues. It was determined that low-grade dysplasias that would progress had the genomic patterns that resembled those observed in high-grade dysplasias (Figure 4), exhibiting increased genomic instability before phenotypic or histological appearances. In contrast, these genetic alterations were rare in benign low-grade dysplasias that did not progress [101, 105]. Another frequent genomic alteration present in oral dysplasias that have a high risk of progression was DNA amplification, which is often an indicator of tumor aggressiveness. Specifically, coamplification of the oncogenes *EGFR* and *CCND1* was observed [101, 105, 106].

This pattern of genomic instability was informative, but it is useful to derive the mechanism of cancer progression. To decipher what pathways were disrupted and what genes were involved, the aCGH data was overlaid with publically available expression data. It was discovered that the majority of genes altered at the genomic and transcriptomic level converged into a single pathway, the FGF signalling pathway. Oral cancer is therefore an example of the importance of considering the downstream effect of genomic alterations and determination of biochemical pathways affected. Pathways may be affected early or late, but the ultimate phenotype may be redundant.

### 3.2. Lineage-Specific Genetic Features in Lung Cancer

Lung cancer is divided into two major categories, small cell lung cancer (SCLC) and nonsmall cell lung cancer (NSCLC). The latter accounts for 85% of lung cancer cases and is further subdivided into adenocarcinoma (AC), squamous cell carcinoma (SqCC), and large cell carcinoma (LCC). While the prevalence of AC is rising, SqCC accounts for approximately 30% of lung cancer cases [107, 108].

The differential response to specific therapy highlights the importance of treating lung cancer subtypes as genetically distinct. For example, Gefitinib and Erlotinib target the tyrosine kinase surface receptor, EGFR, which was found to be overexpressed in at least 50% of lung cancers [109]. Strikingly, clinical trials revealed that most patients with NSCLC had moderate to no drug response, except only a subset of patients that were highly responsive [110–113]. The responders had mutations in the catalytic domain of *EGFR*, which resulted in increased drug efficacy [114]. Specifically, *EGFR* mutations and drug sensitivity were associated with the AC subtype, never-smoking status, Asian ethnicity, and female gender [110]. In another example, drug response variability was seen with the advent of the thymidylate synthase (TS) inhibitor, Pemetrexed. Pemetrexed exhibits a higher efficacy in AC compared to SqCC, likely due to the generally higher levels of TS expression in SqCC [115–117]. These clinical results have provided an impetus to delineate the different genetic mechanisms governing lung cancer subtypes, in order to tailor treatment for SqCC and AC. 

**Figure 5:**
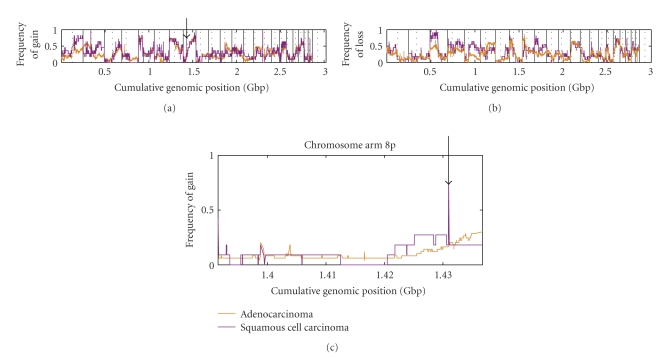
Genome-wide comparison of lung adenocarcinoma (AC) and squamous cell carcinoma (SqCC). Affymetrix SNP 6.0 data for 11 SqCC and 49 AC cell lines were downloaded from the Wellcome Trust Sanger Cancer Genome Project. Data were analyzed against a pool of a normal reference samples using Partek Genomics Suite. For each position in the genome, the frequency of (a) gain and (b) loss are shown for SqCC (purple) and AC (orange). (c) Magnified view of chromosome arm 8p shows that SqCC has a higher frequency of segmental DNA gain at cumulative position 1.43 Gbp as compared to AC.

Evidence from lung tumors is rapidly accumulating to indicate not only that are AC and SqCC composed of different genomic backgrounds (Figure 5), but also that they arise from the disruption of different biochemical pathways. Two key lineage-specific genetic differences between SqCC and AC were recently discovered using integrative genomics technologies. Both genes appear to be specific to the SqCC lineage and were found to be highly amplified. These genes were *SOX2,* likely involved in squamous pluripotency and differentiation [118], and *BRF2*, a RNA polymerase III transcription initiation factor controlling cell growth [119]. These discoveries suggest that SqCC and AC are genetically distinct.

### 3.3. Genes and Pathways Disrupted in Squamous Cell Carcinoma of the Skin

Like lung cancer, skin cancer is divided into two types: melanoma skin cancer and nonmelanoma skin cancer (NMSC). Skin SqCC is a subtype of nonmelanoma skin cancer (NMSC), a classification that also includes basal cell carcinoma (BCC). The standard treatment option for NMSC is surgical biopsy, which has been applied frequently and successfully without recurrence [120]. Occasionally, NMSC develops to an advanced stage, and in 3%–5% of cases the tumor metastasizes, lowering the 5-year survival rate to 14%–39% [121]. Advanced cases of NMSC are not always treatable by biopsy alone [120]. Subsequent treatment options include chemotherapy, radiation therapy, or a combination of surgery and radiation [120, 121]. To date, chemotherapeutics such as cisplatin, 5-Fluor-Uracil, Paclitaxel, and combinations have been utilized with limited success [121]. Knowledge of the molecular mechanisms controlling NMSC tumors holds promise in improving treatment.

SqCC and BCC should be regarded as genetically distinct as different biological pathways are disrupted in these disease types [122]. The pathogenesis of SqCC is complex and dependent on many intrinsic and extrinsic factors. It is generally recognized that the most important extrinsic factor is UV sunlight exposure, as incidence of squamous cell carcinoma increases with lifetime UV exposure [123]. Using previously unexposed human skin, exposure to UV was shown to result in a missense point mutation with a UV signature in one *TP53* allele, and the remaining allele is deleted [124]. SqCC undergoes the inactivation of multiple tumor suppressor genes, namely, *TP53* which is inactivated in 90% of all precancerous lesions and invasive tumors, and also *P16* and *P14* [125, 126]. *TP53* is also mutated in BCC; however, the mutation rate decreases from 90% to 50% [127–129]. Broader examination of subtype-specific changes indicates that SqCC and BCC accrue alterations of different biochemical pathways.

**Figure 6:**
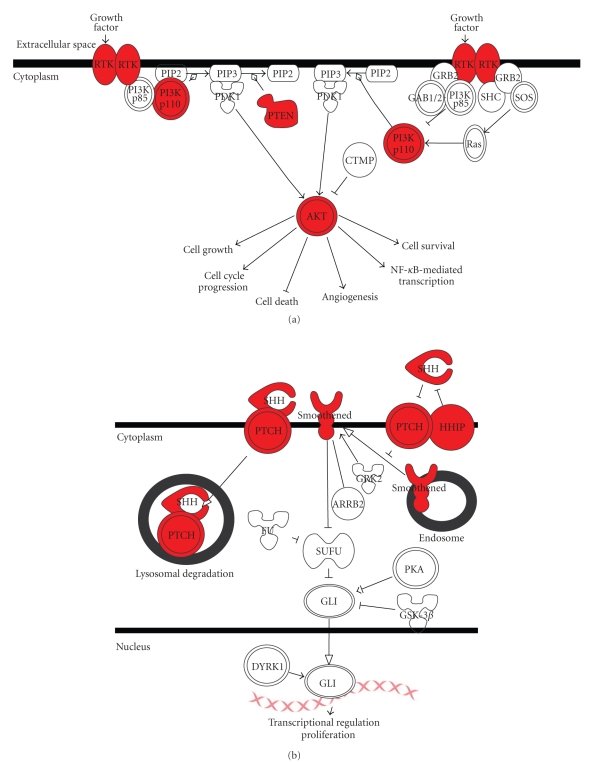
Major pathways affected in NMSC. (a) SqCC undergoes frequent alterations to the PI3K/AKT signaling pathway. (b) BCC undergoes frequent alterations to the sonic hedgehog pathway. Various pathway components affected are highlighted in red.

In SqCC, deregulation of the PI3K/AKT signaling pathway is common. This pathway has been demonstrated to control cellular functions such as apoptosis, cell proliferation, and cell growth in isogenic cell lines [121, 130]. Mechanistically, the most common mechanisms of alteration are constitutive growth factor receptor activation, *PI3K *amplification or mutation, *AKT* amplification or mutation, and *PTEN* inactivation (Figure 6(a)) [121]. The main affected pathway in BCC is the sonic hedgehog (Shh) signaling pathway. In skin, this pathway regulates the development of follicles and sebaceous glands, as well as maintains stem cell populations [131]. In tumors the *patched* (*PTCH*) or *smoothened* (*Smo*) genes have been found to be most commonly altered, with mutation rates of 30%–60%. *PTCH* is inactivated most commonly by chromosomal deletion or by mutation [131, 132], while mutations in *sonic hedgehog* (*Shh*) and *Smo* have also been documented. In addition to genomic alterations, expression level analysis has revealed increased levels of *PTCH* and *hedgehog interacting protein* (*HIP*) mRNA, adding a dimension of change (Figure 6(b)). Much like oral cancer, both SqCC and BCC experience dysregulation of a certain pathway, via disruption of multiple component genes.

SqCC is associated with significant chromosomal aberrations at all stages of progression (reviewed in [133, 134]). In general, SqCC has increased genomic instability with 25%–90% of tumors demonstrating DNA aneuploidy. In contrast, BCC genomes are reasonably stable demonstrating 9%–40% aneuploidy. Early studies examined the extent of LOH in SqCC, finding it to be both diverse and widespread. Frequent regions of LOH were determined at 13q (46%), 9p (41%), 17p (33%), 17q (33%), and 3p (23%) [135]. In addition, SqCC has a range of other chromosomal alterations, including gain of chromosomes 3q, 8q, 14q, and 17q and deletion of chromosomes 8p, 9p, 17p, and 18q [133]. In BCC, loss of genetic material was found to be confined to 9q22.3, the locus of the *PTCH* tumor suppressor gene [136–138]. A more complete picture of the genes and pathways disrupted in SqCC development could be obtained if these genomic data were integrated with epigenetic and transcriptomic data sets. However, the literature on genome-wide scans for epigenetic alterations and transcriptional changes in NMSC is scarce; global approaches will be crucial to identifying novel genes and pathways involved in the carcinogenesis of NMSC.

## 4. Squamous Cell Carcinoma and Integrative Genomics: Present and Future

**Figure 7:**
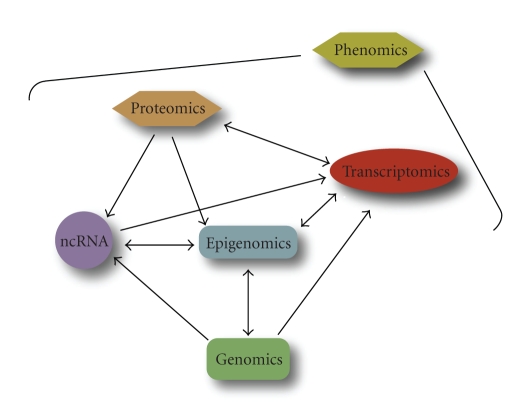
The integrated network of genome-wide analyses comprising the cancer omics landscape. Phenomics or phenotypic effects are the direct consequence of the perturbations of genes and pathways which could be traced to genetic and epigenetic alterations when multiple dimensions of information are integrated to connect upstream causes with downstream effects.

While genetic, epigenetic, and transcriptional changes are associated with disease, their pathological impact is ultimately held at the levels of protein, cell, tissue, and organismal functions. It is widely believed that integrative phenomics—high throughput phenotyping of any type—will reveal different cancer subtypes with distinct biochemical and biological profiles (Figure 7). After transcriptomics, proteomics and metabolomics were the next types of phenomics to be developed [139]. 

The proteome is the universe of proteins, and proteomics is the study of the structure, regulation, and function of the proteome. The most obvious types of knowledge to be gained are the functional consequences of differential protein expression, profiles of proteins and their posttranslational modification (phosphorylation, ubiquitination, acetylation, palmitoylation, polysialylation, proteolytic cleavage, and dozens more), and identification of protein-protein interactions. Recent reviews address different aspects of cancer proteomics [139–142]. The most common proteomics approach has been the coupling of two-dimensional gel electrophoresis with mass spectrometry (MS). This gel methodology can resolve up to 11000 spots but more commonly separates approximately 2000–4000. 2D fluorescence difference gel electrophoresis (2-D DIGE) uses ultrasensitive fluorescent dyes to label multiple samples in different colors, thus allowing the relative quantification of the same proteins from different samples. More recently, gel-free proteomic techniques have been developed, such as multidimensional protein identification technology (MudPIT). There have been many recent developments in MS sample preparation, methodologies, and data analysis [143]. Another large-scale approach to detect proteins is antibody microarrays [144]. The most common techniques used to discover protein-protein interactions are affinity purification coupled with MS and yeast two-hybrid analysis [145]. Among the most important new applications of proteomics are those defining the universe of posttranslational modifications (e.g., related to signaling or metabolism) and conducting functional screens of gene (e.g., using shRNA) and compound libraries. Other recent studies have begun to yield candidate biomarkers and suggest therapeutic pathways. 

Metabolomics is the large-scale identification and quantification of metabolites [146]. These include sugars, salts, amino acids, peptides, lipids, acids, bases, steroid hormones, and so forth. There are almost 7000 known endogenous metabolites from 52 classes of compounds [147], but there are likely many more to be discovered. Metabolites are not only the products of cellular metabolism but also signaling molecules involved in the regulation of physiological states (e.g., feedback regulation, homeostasis). Thus, metabolomics may not only lead to the discovery of new biomarkers, but is also likely to reveal altered biochemical and physiological states. That would, in turn, suggest biochemical pathways that could be targeted therapeutically, and cellular assays to screen drugs or biologicals (e.g., siRNA, humanized antibodies). The most common approaches for global metabolomic profiling involve different separation technologies—such as liquid/gas chromatography and capillary electrophoresis—coupled with detection and identification using MS or nuclear magnetic resonance spectrometry (NMR) [148]. The data generated in this field is complex, and there is intense focus on the development of statistical methods [149] and database resources [150]. Oncology is one of the leading applications of metabolomics, both in clinical [151] and animal studies [152]. A major area of recent interest in cancer metabolomics is that related to the Warburg effect—the switch from oxidative phosphorylation to glycolysis that is associated with many cancers [153], including OSCC [154]. The state of the art in cancer metabolomics is illustrated by a prostate study that profiled 1126 metabolites across 262 clinical samples from 42 tissues and 110 each of urine and plasma [155]. The resulting profiles distinguished pathological subtypes (as well as the benign condition) and identified sarcosine as a metabolite that is highly increased with prostate cancer progression.

Many areas of proteomics and metabolomics have unique methodological requirements and are considered subdisciplines—for example, glycomics, lipidomics, spliceosomics, phosphoproteomics, and ubiquinomics. A major challenge for human studies is that available tissues are generally limited to biopsy samples, circulating blood cells (including purified lymphocytes, hematopoietic stem cells, platelets, etc.), and body fluids [142]. Recently, methodologies have been developed to allow analysis of not only fresh and frozen tissues but also formalin-fixed and paraffin-embedded tissue. Notably, workflows are being developed to collect and process diverse clinical samples for integrative omics studies [140].

In conclusion, from the early studies integrating genomic and epigenomic data, it is apparent that much can be gained when a multifaceted approach is employed. With multiple large-scale initiatives prioritizing this style of analysis on a diverse spectrum of tumor types, and with the continuing evolution of high throughput technologies to measure additional dimensions, the ability to perform bona fide molecular systems biology in clinical specimens may finally be in sight.

## Figures and Tables

**Table 1:** Genome-wide methods for identifying genetic alterations.

Alteration	Method of genome-wide analysis	References
Chromosomal translocation	Spectral karyotyping (SKY)	[8–12]
DNA copy number	Array comparative genomic hybridization (aCGH)	[13–17]
	Digital karyotyping and genome sequencing	
Loss of heterozygosity	Single-nucleotide polymorphisms (SNPs) analysis	[18, 19]
Mutation	Gene-specific, exomic, and whole-genome sequencing	[20–23]

**Table 2:** Techniques for genome-wide analysis of the methylome.

** Bisulfite Conversion**. Sodium bisulfite treatment converts unmethylated cytosines to uracil, while methylated cytosines remain unaffected. This change in sequence can be discriminated using the techniques listed here, and relative methylation can be quantified relative to reference DNA.	
Applications coupled with bisulfite conversion	References

Whole-genome sequencing	[40]
Illumina platforms	[41]
Bisulfite microarray (BS chip)	[41]
Methylation-specific quantum dot fluorescence resonance energy transfer (MS-qFRET)*	[42]
Bisulfite padlock probes (BSPPs)	[43]

**Methylated DNA Precipitation**. DNA is fractionated and methylated DNA sequences are subsequently eluted through the use of specific antibodies or methyl binding proteins. Following completion of the applications listed below, precipitated DNA can be analyzed using CpG island or promoter microarray hybridization, or sequenced.	

Applications Coupled with Methylated DNA Precipitation	References

Methylated DNA immunoprecipitation (MeDIP) and (mDIP).* Methylation-specific antibodies are utilized to immunoprecipitate methylated DNA fragmented by sonication. *	[26, 34, 44]
Comprehensive high throughput arrays for relative methylation (CHARM). *Custom tiling array used in conjunction with a genome-weighted smoothing algorithm. *	[45]
*Antibodies specific to 5-methyl-cytosine or methyl binding proteins are used to immunoprecipitate fragments of methylated DNA.*	[27]
Methylated-CpG island recovery assay (MIRA). *A matrix of methyl binding proteins is used to elute methylated DNA. *	[46]

**Methylation-sensitive Enzymes**. The sensitivity of certain restriction endonucleases (REs) to DNA methylation is exploited to differentially digest DNA.	

Applications coupled with restriction enzymes	References

Methyl-sensitive cut counting (MSCC)	[43]
*Hpa*II tiny fragment enrichment by ligation-mediated PCR (HELP) differentially amplifies methylated DNA	[47]
Restriction landmark genome scanning for screening methylated sites (RGLS-M)	[48]
Comprehensive high throughput arrays for relative methylation (CHARM)	[46]

**Table 3:** Software for integrative analysis of Multidimensional omics data.

Program	Application	Website	Reference
Agilent Genomic Workbench 5.0	Genomics	http://www.chem.agilent.com/en-us/products/instruments/dnamicroarrays/dnaanalyticssoftware/pages/default.aspx	
	Epigenomics		n/a
	Transcriptomics		
SIGMA2	Genomics	http://www.flintbox.com/technology.asp?page=3716	
	Epigenomics		[85, 86]
	Transcriptomics		
Integrative Genomics Viewer	Genomics Transcriptomics	http://www.broadinstitute.org/igv/	n/a
Nexus Copy Number	Genomics Transcriptomics	http://www.biodiscovery.com/index/nexus	n/a
CGH Fusion	Genomics Transcriptomics	http://www.infoquant.com/index/cghfusion	n/a
ISA-CGH	Genomics Transcriptomics	http://www.isacgh.bioinfo.cipf.es	[87]
VAMP	Genomics	http://www.bioinfo-out.curie.fr/projects/vamp/	
	Epigenomics		[88]
	Transcriptomics		
Partek Genomics Suite	Genomics	http://www.partek.com/partekgs	
	Epigenomics		n/a
	Transcriptomics		
	Genomics	http://www.GenomicsPortals.org/	
Genomics Portals	Epigenomics		[89]
	Transcriptomics		
CHESS	Genomics Transcriptomics	http://www.biostone.khu.ac.kr/CHESS	[90]
integrOmics	Genomics	http://www.CRAN.R-project.org/	
	Epigenomics		[91]
	Transcriptomics		
SEURAT	Genomics	http://www.seurat.r-forge.r-project.org/	
	Epigenomics		[92]
	Transcriptomics		
Genome Studio	Genomics	http://www.illumina.com/software/genomestudio_software.ilmn	
	Epigenomics		n/a
	Transcriptomics		
